# Ethnic differences in meat consumption attitudes, norms and behaviors: A survey of White, South Asian and Black ethnic groups in the UK

**DOI:** 10.1016/j.appet.2024.107359

**Published:** 2024-04-16

**Authors:** Elif Naz Çoker, Rachel Pechey, Susan A. Jebb

**Affiliations:** Nuffield Department of Primary Care Health Sciences, University of Oxford, Radcliffe Primary Care Building, Radcliffe Observatory Quarter, Woodstock Road, Oxford, OX2 6GG, UK

**Keywords:** Meat consumption, Ethnicity, Gender, Socioeconomic status, Norm perception

## Abstract

A reduction in meat consumption is necessary to mitigate negative impacts of climate change and adverse health outcomes. The UK has an increasingly multi-ethnic population, yet there is little research on meat consumption habits and attitudes among ethnic groups in the UK. We ran a survey (N = 1014) with quota samples for ethnic groups and analyzed attitudes, behaviors and norm perceptions of White, South Asian and Black British respondents. Most respondents believe overconsumption of red and processed meat has negative impacts on health (73.3%) and the environment (64.3%).South Asian respondents were statistically significantly less likely to be meat eaters than White respondents (OR = 0.44, 95% CIs: 0.30-0.65, t = −4.15, p = 0.000), while there was no significant difference between White and Black respondents (OR = 1.06, 95% CIs: 0.63–1.76, t = 0.21, p = 0.834). Both South Asian (OR = 2.76, 95% CIs: 1.89–4.03 t = 5.25, p = 0.000) and Black respondents (OR = 2.09, 95% CIs: 0.1.30–3.35, t = 3.06, p = 0.002) were significantly more likely to express being influenced by friends and family in their food choices than White respondents. South Asian (OR = 3.24,95% CIs: 2.17–4.84, t = 5.74, p = 0.000) and Black (OR = 2.02,95% CIs: 1.21–3.39, t = 2.69, p = 0.007) respondents were also both significantly more likely to report they would want to eat similarly to their friends and family than White respondents. Statistical analyses suggested some gender and socioeconomic differences across and among ethnic groups, which are reported and discussed. The differences in meat consumption behaviors and norm conformity between ethnic groups raises the prospect that interventions that leverage social norms may be more effective in South Asian groups than Black and White groups in the UK.

## 1 Introduction

Livestock production has a significant negative impact on the environment (Gerber et al., 2013; Scarborough et al., 2023) and excess consumption of animal products is associated with a number of negative health outcomes (Ekmekcioglu et al., 2018; Papier et al., 2021). Despite this, the UK population still eats 219 g of meat per day on average (Our World in Data), which is about three times more than recommended by the EAT Lancet Commission on Planetary Health (Willett et al., 2019).

In Western countries, knowledge of the impacts of overconsumption of red and processed meat on environment is still limited and many do not consider that reducing meat consumption as a pro-environmental behaviour (Hartmann & Siegrist, 2017; Macdiarmid et al., 2016; Sanchez-Sabate & Sabaté, 2019; van Bussel et al., 2022). Additionally, the majority of people who are omnivores believe that eating meat is “natural, necessary, nice and normal” (Piazza et al., 2015). Interventions to reduce meat intake need to overcome these established attitudes.

However, it would be an overgeneralization to assume that there are no differences in dietary habits and food choices between different ethnic groups, especially among those with cultural practices and traditions around food that may have been influenced by countries with historically lower meat consumption levels (e.g. South Asian, African, and Caribbean countries). There is currently relatively little to no quantitative data and research on these (Leung & Stanner, 2011), due to sample sizes in nationally representative surveys being too small to make precise estimates (Leahy et al., 2010; Stewart et al., 2021), and researchers citing problems of lack of engagement and language barriers in gathering data from minority ethnic groups (Chowbey & Harrop, 2016). Only a few studies provide qualitative evidence regarding the eating habits of ethnic minority populations in the UK suggest that these groups have different diet patterns and point to sociocultural factors that may result in different food choices, including having an increased likelihood of following religious restrictions for diet, sharing meals, living in multigenerational households, and consuming a diet that is a mixture of their ethnic cuisine and foods from where they currently live (Gilbert & Khokhar, 2008). For example, a case study of interviews with Pakistani and Gujarati mothers living in north of England found that food had a distinct social and religious value attached to it which was separate from its nutritional value and the importance of eating with community was highlighted (Chowbey & Harrop, 2016). A focus group study with 110 parents in Luton found that both Black and South Asian parents want to bring their children up on a culturally traditional diet, and emphasized the role of cooking and eating with grandparents in promoting these traditional food choices (Cook et al., 2021).

Eating is indeed a social behavior, and there is a strong body of evidence that shows people are influenced by the behaviors and expectations of others when making food choices (Robinson et al., 2014; Stok et al., 2016). Often described as ‘unwritten rules of society’, social norms refer to the approved and expected ways of behaving in a given context (Cialdini et al., 1990; Fehr & Fischbacher, 2004). Social norms have been proven to be influential in different eating contexts like increasing fruit and vegetable intake, reducing meat consumption, preference of healthy snacks etc. (Collins et al., 2019; Gonçalves et al., 2021; Huitink et al., 2020; Mollen et al., 2013; Robinson et al., 2013; Sparkman & Walton, 2017; Thomas et al., 2017).

Social norms around meat consumption can also inform expectations and acceptance of behaviors of certain demographic groups. For instance, a number of studies have shown that people think high levels of meat consumption are associated with masculinity whereas vegetarianism is seen as feminine (Rozin et al., 2012; Ruby & Heine, 2011). Indeed, evidence shows that on average, men consume more meat than women and see it as an integral part of their diet while women express more openness to reducing their consumption (de Boer et al., 2017; Hinrichs et al., 2022; Rothgerber, 2013; Rozin et al., 2012).

The relationship between socioeconomic status (SES) and meat consumption seems more complex compared to gender. While meat consumption has historically been viewed as a symbol of affluence and financial status, recent research has found an “inverted-U″ relationship between SES and meat consumption, where consumption first increases as people get more affluent, and then decreases for the top proportion of high SES individuals (Clonan et al., 2016; Maguire & Monsivais, 2015).

With little research having been done on social norms of meat consumption with regard to its cultural significance for different ethnic groups (Ellithorpe et al., 2022), it is important to explore how ethnic groups differ in their attitudes, beliefs and normative perceptions around meat consumption. In order to better understand these cultural differences, it is also important to explore whether and in which direction the associations between meat, gender, and SES change across different ethnic groups.

### 1.1 Research aims

The present survey study aimed to explore differences between White, South Asian and Black ethnic groups in the UK in terms of their.

a meat consumption patterns
b intentions to change meat consumption
c beliefs about the impact of overconsumption of meat on personal health and the environment
d perceptions of influence and support from their friends and family regarding food choices.

The secondary aim was to examine interactions between ethnicity and gender and socioeconomic status (SES) for the same measures and observe whether different patterns emerged for White, South Asian, and Black ethnic groups.

## 2 Methods

### 2.1 Procedure

The survey took place as a part of a joint study with Reuters Institute for the Study of Journalism, University of Oxford. YouGov, a British international market research company, was employed for the recruitment of participants and the administration of the survey and data collection. Ethics approval was obtained from the University of Oxford Central University Research Ethics Committee with reference number: R42505/RE002.

The initial sample was recruited (N = 1244) and data was collected between February 23rd and March 2nd, 2021. Upon detecting an error in the age quota, additional participants (N = 200) were recruited and their data was collected between May 11th and May 14th, 2021.

### 2.2 Sample population

The sample for White British respondents were recruited separately from the sample for all other ethnic groups combined due to different stratification strategies for each sample. Both samples came from a previously selected panel of around 1,000,000 UK respondents who are either citizens or individuals ordinarily residing in the UK. The ethnic minority groups sample was stratified based on region, age, gender, ethnicity, work status and country of birth, while the White sample was stratified based on age, gender, education status and social grade in order to ensure national representation. Upon the detection of an error with the age quotas for the White respondents and discussions with the market research company, additional individuals were recruited in the second wave to ensure the sample was representative of the current UK population estimates.

### 2.3 Measures

#### 2.3.1 Demographics

Participants were asked to provide information on their gender, age, social grade, education level, and ethnicity (see Table 1).

#### 2.3.2 Survey questions

Participants were asked to answer questions relating to their meat eating habits and dietary patterns, intentions to change their meat consumption, beliefs around the health and environmental impact of overconsumption of meat, perceived influence of close friends and family, importance of eating similarly to friends and family and anticipated support of friends and family to reduce meat consumption (see Table 2 in section 3. Results for exact wording of each question). The measures were developed as novel, single-item measures that were worded to be clear, concise, and capturing distinct concepts and were based on measures used in previous related research (Çoker, Jebb, et al., 2022).

### 2.4 Data analysis

Respondents were asked to provide ethnicity data choosing from one of the ethnic subgroups as outlined in the 2011 Census of England and Wales (UKGovernment, 2022) From this detailed subgroup level data, and following the most recent ethnicity group categorizations in the 2021 Census for England and Wales, we have grouped Caribbean, African and other Black and Black British respondents into the “Black or Black British” category. Again, in line with Census categorization, English, Welsh, Scottish, Northern Irish, Irish, Gypsy or Irish Traveller and other White respondents were grouped into the “White or White British” category. While the Census categorizes all Asian backgrounds into one group, we grouped Indian, Pakistani, Bangladeshi respondents into the “South Asian or South Asian British” category and did not include Chinese and other Asian or Asian British respondents (N = 204) in this category in order to capture the significant cultural differences with regards to eating habits and meat consumption between the Indian Subcontinent and East Asia. All mixed or multiple ethnic background respondents were grouped into the “Mixed and multiple ethnic background” (N = 100) group and Arab and other ethnic backgrounds not listed as an option were all grouped into a “Other ethnic group” (N = 126), again in line with Census 2021 categorizations.

Our analyses only included White (N = 403), South Asian (N = 382), and Black (N = 229) groups because these constituted the only three groups that both had enough respondents to make the analyses statistically adequately powered and we could be confident enough that they represented relatively culturally homogenous groups. The other ethnic groups did not have enough numbers (e.g. Chinese (N = 42) and Arab (N = 23) groups) or were too heterogeneous (e.g. “Mixed” group including individuals with undisclosed mixed ethnic backgrounds) to be meaningfully included in the analysis. After these exclusions, our final sample consisted of 1014 participants.

We have measured socio-economic position (SEP) of participants through their education level and occupation-based social grade. Since there was a considerable overlap between these two measures, we have analyzed differences in responses based on social grade only, categorizing respondents into one of the two groups: lower-SEP and higher-SEP.

The data was analyzed descriptively using STATA (16.1, StataCorp LLC). Survey weights were set using the weights assigned by YouGov, who collected the raw data on behalf of the researchers, in order to account for demographic differences between ethnic group samples and the panel population they were recruited from in order to improve the sample’s representativeness. For descriptive analyses, answers from 5-point Likert scale questions were collapsed into 3-points (agree, don’t know, disagree) for easier interpretation. Only for the analysis of the item that measured intentions to change meat consumption, those who already did not eat meat and did not intend to change this were excluded from this specific analysis (N = 390). Statistical analyses were conducted via logistic regressions. For these, a dummy for the dependent variable was created so that “strongly agree” and “agree” answers were collapsed into one category, and all other answers were collapsed into another, enabling us to regress the likelihood of agreeing with a statement on ethnicity, gender, and social grade. For the diet identity measure, we ran two separate analyses: one for comparing the likelihood of being vegetarian or vegan against other options (pescatarian, flexitarian, and meat-eater), and the other comparing the likelihood of being a meat-eater against all others. This was done to capture nuances between completely excluding all types of meat from the diet versus different kinds of meat-eating (e.g. eating only fish and seafood, or eating all meat except pork). Since our study was hypothesis *generating* and not hypothesis *confirming*, we did not have pre-specified reference categories for ethnicity, gender or SEP. We therefore decided to have the White ethnicity, men, and higher-SEP as our reference categories when running the regressions. Subgroup analyses for the role of gender and SEP were run in separate regressions for each ethnic group.

## 3 Results

### 3.1 Results by ethnicity

#### 3.1.1 Diet and meat consumption

**Fig. 1:**
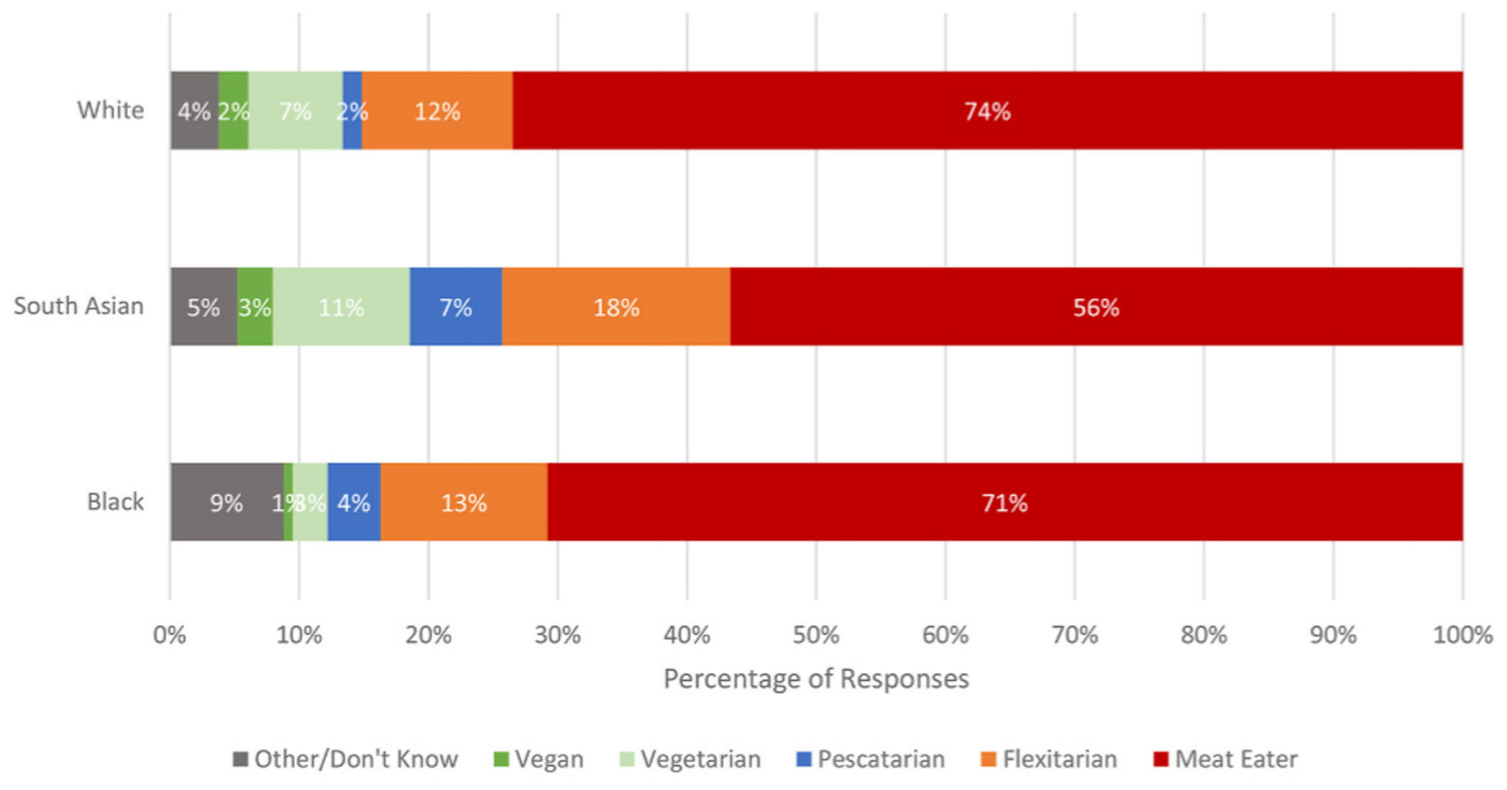
Meat consumption dietary patterns by ethnicity.

Slightly more than half of respondents with South Asian ethnicity were meat eaters while about two thirds of White and Black respondents followed an omnivore diet (see Fig. 1). South Asian respondents were statistically significantly less likely to be meat eaters than White respondents (OR = 0.44, 95% CIs: 0.30-0.65, t =− 4.15, p = 0.000), while there was no significant difference between White and Black respondents (OR =1.06, 95% CIs: 0.63–1.76, t =0.21, p =0.834). However, there were no statistically significant differences between ethnic groups in their likelihood in being vegetarian or vegan, further emphasizing that the differences were between flexitarians and pescatarians.

#### 3.1.2 Reduction intentions

South Asian participants were more likely than White respondents to want to decrease or to have recently decreasedtheir meat consumption (OR =1.82, 95% CIs: 1.24–2.67, t =3.07, p = 0.002), but no significant difference was observed between White and Black participants. Both South Asian (OR = 6.46, 95% CIs: 2.60–16.02, t = 4.03, p = 0.000) and Black (OR = 5.89, 95% CIs: 2.06–16.87, t = 3.31, p = 0.001) respondents were significantly more likely than White respondents to want to increase or to have recently increased their meat consumption.

#### 3.1.3 Meat and health

The majority of the population (73.3%) agreed that overconsumption of red and processed meat has a negative impact on health. Compared to White respondents, South Asian (OR = 1.19, 95% CIs: 0.78–1.80, t = 0.8, p = 0.422) and Black respondents (OR = 1.17, 95% CIs: 0.72–1.91, t = −0.62, p = 0.553) were not statistically significantly more likely to agree with this statement.

#### 3.1.4 Meat and environment

The majority of the population (64.3%) agreed that overconsumption of red and processed meat has a negative impact on the environment. Compared to White respondents, South Asian (OR = 1.24,95% CIs: 0.85–1.81, t = 1.13, p = 0.260) and Black respondents (OR = 0.93, 95% CIs: 0.55–1.55, t = −0.29, p = 0.771) were not statistically significantly more likely to agree with this statement.

#### 3.1.5 Support for reducing meat consumption

The majority (72.5%) of total respondents believed that they would have the support of their friends and family if they decided to change their meat consumption. Compared to White respondents, neither South Asian (OR = 1.13,95% CIs: 0.77–1.68, t = 0.63, p = 0.530) nor Black respondents (OR = 1.32, 95% CIs: 0.69–2.55, t = 0.83, p = 0.405) differed in their likelihood of expecting support.

#### 3.1.6 Influence of friends and family in food choices

**Fig. 2:**
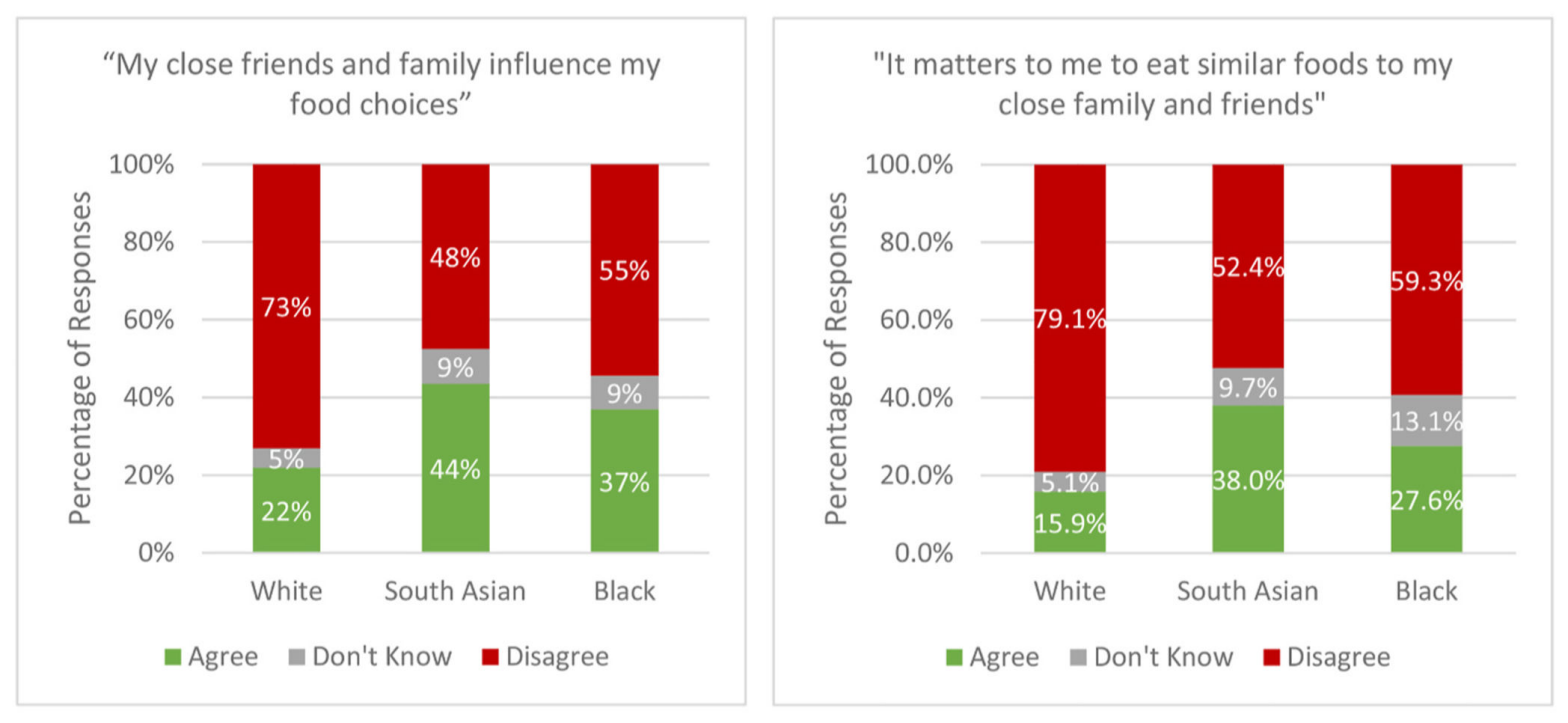
a. and b. Perception of influence and importance of eating similarly to eat friends and family by ethnicity.

Higher proportions of respondents of Black (36.9%) and South Asian (43.5%) ethnicity agreed that their family and friends influenced their food choices (See Fig. 2a), while White respondents were less likely to agree with this statement (21.9%). Statistical analyses also demonstrated that compared to White respondents, both South Asian (OR = 2.76, 95% CIs: 1.89–4.03 t = 5.25, p = 0.000) and Black respondents (OR = 2.09, 95% CIs: 0.1.30–3.35, t = 3.06, p = 0.002) were significantly more likely to express being influenced by friends and family in their food choices.

#### 3.1.7 Similarity to friends and family in food choices

Higher proportions of respondents of Black (27.6%) and South Asian (38.0%) ethnicity agreed that their family and friends influenced their food choices (See Fig. 2b). Respondents of South Asian ethnicity were significantly more likely to report they would want to eat similarly to their friends and family than White respondents (OR = 3.24,95% CIs: 2.17–4.84, t = 5.74, p = 0.000). A similar trend was observed for Black respondents, who also were significantly more likely express a desire to eat similarly to friends and family compared to White respondents (OR = 2.02,95% CIs: 1.21–3.39, t = 2.69, p = 0.007).

### 3.2 Role of gender and socioeconomic status in meat consumption behaviors, beliefs and norms across different ethnic groups

#### 3.2.1 Diet and meat consumption

Descriptive analyses suggest across all ethnicities, women were more likely to follow non-omnivore diets than men, this difference was especially marked in the White ethnic group (81.6% versus 66.2%). Women across all ethnicities were also more likely to identify as being flexitarian than men. (see Table 3). Logistic regression analysis showed that in the overall population, women were statistically significantly more likely to be vegan or vegetarian than men (OR = 2.52, 95% CIs: 1.41–4.52, t = 3.12, p = 0.002). Analyses within ethnic groups showed that White women were statistically significantly more likely to be vegan or vegetarian than White men (OR = 3.05, 95% CIs: 1.33–6.96, t = 2.65, p = 0.008), but the differences within South Asian and Black ethnic groups were statistically non-significant. Similar patterns emerged for meat-eating such that women in the overall population were statistically significantly less likely to be meat-eaters (OR = 0.38, 95% CIs: 0.22-0.64, t = − 3.61, p = 0.000), while there were no gender differences within the other two ethnic groups.

In the overall sample, there were no statistically significant differences between lower-SEP and higher-SEP respondents in their likelihood of being vegan or vegetarian (OR = 0.56, 95% CIs: 0.28–1.08, t = −1.73, p = 0.084) or being a meat-eater (OR = 0.55, 95% CIs: 0.28–1.08, t = −1.73, p = 0.084). Both White (OR = 1.95, 95% CIs: 1.15–3.31, t = 2.48, p = 0.014) and South Asian (OR = 2.17 95% CIs: 1.08–4.35, t = 2.20, p = 0.029) lower-SEP respondents were statistically significantly more likely to be meat-eaters compared to their higher-SEP counter-parts, while there was no significant difference within the Black ethnic group. Within ethnic groups there was no statistical difference between lower- and higher-SEP respondents for any the three groups in terms of likelihood of being vegetarian or vegan (see Table 4).

#### 3.2.2 Reduction intentions

There were no statistically significant differences between men and women in the overall sample in either wanting to increase or decrease their meat consumption. Women of South Asian ethnicity were more likely to want to increase or to have recently increased their meat intake than their male counterparts (OR = 2.85, 95% CIs: 1.11–7.37, t = 2.18, p = 0.030). Contrastingly, White women were more likely to want to decrease or to have recently decreased their meat intake than their male counterparts (OR = 1.79 95% CIs: 1.12–2.86 t = 2.43, p = 0.015).

In the overall sample, there were no statistically significant differences between lower-SEP and higher-SEP respondents in their likelihood of either increasing or decreasing their meat consumption. A greater proportion of lower-SEP respondents of South Asian ethnicity reported recently having decreased or wanting to decrease their meat consumption (60.3% versus 41.0%) while more higher-SEP respondents said they wanted an increase or recently have increased (11.9% versus 5.8%). However, analyses within ethnic groups showed that there was no statistical difference between higher- and lower-SEP respondents for any of the three groups.

#### 3.2.3 Meat and health

There were no statistically significant differences between men and women in the overall sample in their likelihood of agreeing that overconsumption of red and processed meat had negative health impacts. There was no statistical difference between men and women’s responses to this item within any ethnic group.

Lower-SEP respondents were less likely to believe that overconsumption of red and processed meat was unhealthy compared their higher-SEP counterparts in the total sample (OR = 0.62, 95% CIs: 0.42-0.91, t = −2.45, p = 0.014). This difference was seen among the White (OR = 0.51, 95% CIs: 0.32-0.82, t = −2.79, p = 0.005) and South Asian (OR = 0.38, 95% CIs: 0.19-0.78, t = −2.66 p = 0.008) ethnic groups, but not for Black respondents (OR = 2.41, 95% CIs: 0.99–5.91 t = 1.94, p = 0.053).

#### 3.2.4 Meat and environment

Across all ethnicities, women had a higher likelihood of believing meat consumption had negative impacts on the environment than men (OR = 1.55, 95% CIs: 1.08–2.21, t = 2.40, p = 0.017). Within ethnic groups, there was no statistical difference between Black men and women or between White men and women, while a significant difference was observed between South Asian men and women (OR = 2.00 95% CIs: 1.07–3.73, t = 2.19, p = 0.029).

Lower-SEP respondents were less likely to believe that overconsumption of red and processed meat was bad for the environment compared their higher-SEP counterparts in the total sample (OR = 0.63, 95% CIs: 0.43-0.92, t = −2.37, p = 0.018). However, there was no statistical difference between Black lower-SEP and higher-SEP respondents or respondents of South Asian ethnicity, while a significant difference was observed between White lower- and higher-SEP respondents (OR = 0.58 95% CIs: 0.37-0.91, t = −2.38, p = 0.018).

#### 3.2.5 Support for reducing meat consumption

There were no statistically significant differences between men and women in the overall sample in their likelihood of thinking they would be supported by friends and family if they reduced their meat consumption (OR = 1.35 95% CIs: 0.91–2.00, t = 1.49, p = 0.136). There also were no statistically significant differences between men and women within any of the three ethnic groups.

In the overall sample, lower-SEP respondents were less likely to anticipate support from their friends and family compared to higher-SEP respondents (OR = 0.62 95% CIs: 0.41-0.94 t = -2.25, p = 0.025). In analyses within ethnic groups, White lower-SEP respondents anticipated less support from their friends and family compared to higher-SEP respondents (OR = 0.49 95% CIs: 0.31-0.78 t = -3.01, p = 0.003). There were no statistically significant differences within Black and South Asian ethnic groups.

#### 3.2.6 Influence of friends and family in food choices

There were no statistically significant differences between men and women in the overall sample in their likelihood of thinking they were being influenced by friends and family in their food choices (OR = 1.06 95% CIs: 0.75–1.50, t = 0.33, p = 0.744). There also were no statistically significant differences between men and women within any of the three ethnic groups.

In the overall sample, lower-SEP respondents were less likely to agree with the statement that their food choices are influenced by friends and family than higher-SEP respondents (OR = 0.53 95% CIs: 0.36-0.79, t = -3.19, p = 0.001). In analyses within ethnic groups, White lower-SEP respondents anticipated less support from their friends and family compared to higher-SEP respondents (OR = 0.54 95% CIs: 0.32-0.93 t = -2.23, p = 0.026). The differences were not significant for Black and South Asian ethnic groups.

#### 3.2.7 Similarity to friends and family in food choices

There were no statistically significant differences between men and women in the overall sample in their likelihood of expressing a desire to eat similarly to friends and family (OR = 1.03 95% CIs: 0.71–1.49, t = 0.13, p = 0.897). There also were no statistically significant differences between men and women within any of the three ethnic groups.

In the overall sample, lower-SEP respondents were less likely to agree that it is important for them to make food choices similar to those of their friends and family than higher-SEP respondents (OR = 0.56 95% CIs: 0.36-0.86, t = -2.63, p = 0.009). This statistical significance was observed for the White ethnic group (OR = 0.40 95% CIs: 0.21-0.76, t = -2.80, p = 0.005), but not for the Black and South Asian respondents.

## 4 Discussion

We found that South Asian respondents were more likely to follow a low/no meat dietary pattern than White respondents, while no differences were observed between White and Black respondents. A quarter to a third of the sample of all ethnicities had recently decreased their meat intake, while a small but notable proportion of respondents of Black and South Asian ethnicity reported that they recently had or intended to increase their meat intake. Compared to White respondents, a higher proportion participants of other ethnicities reported that their eating behaviours were influenced by family and friends, and that they would want to eat similarly to their close others. Three-quarters of the whole sample anticipated they would have support from close others if they wanted to reduce their meat intake, and there were no differences between ethnic groups for this measure.

The higher proportion of low/no meat-eating behaviours in the South Asian ethnic population likely reflects the religious restrictions both Hindu and Muslim South Asians may have on their diet. Practicing Hindus restrict almost all their meat intake, but Muslims may also have to limit their meat intake due to inaccessibility of halal meat (Hamlett et al., 2008).

In line with a growing body of evidence (Clonan et al., 2016; Maguire & Monsivais, 2015), we also found that women were more likely to follow low/no meat-eating diets and report intentions to reduce their meat consumption compared to men. The differences between genders were most pronounced among White participants, supporting literature that has found wide gender differences in prevalence of vegetarianism in Western countries (Modlinska et al., 2020). In most White ethnic cultures in the UK eating meat is strongly associated with masculinity (De Backer et al., 2020; Graça et al., 2019; Modlinska et al., 2020; Rosenfeld & Tomiyama, 2021). As a result, men may feel more pressure to conform to social norms around meat eating being “masculine”, and may expect less support from their social circles if they engaged in a behavior that goes against expected gender roles (Gal & Wilkie, 2010; Modlinska et al., 2020; Rosenfeld & Tomiyama, 2021; Rothgerber, 2013).

Among White and South Asian respondents, more lower SEP individuals followed meat-eating diets than higher SEP ones. This is in line with previous findings which has shown that in the UK, those in higher occupational roles (e.g. managers) consumed significantly less red meat than those in technical and routine occupations (Clonan et al., 2016). The gap was not seen among Black respondents, however.

In Western countries, the industrial mass production of meat has been a common practice for some time, increasing its abundance and making it easy to access. Previous research has shown that once individuals immigrate to a new country, they are likely to change their food choices and adopt some of the practices of this new country (Gilbert & Khokhar, 2008; Schösler et al., 2015). This might be one of the reasons why we found that a greater proportion of South Asian women indicated a desire to increase their meat consumption, suggesting a change from their traditional, lower-meat diet to a Western, higher-meat one. Contrastingly, we found that White women were more likely to want to decrease their meat consumption. This could be a reflection on recent findings that meat consumption in the UK seems to have peaked in late 2000s, and has been modestly, but gradually decreasing in 2010s ((Stewart et al., 2021).

In contrast to recent literature, we found that the majority of the participants agreed that eating less red and processed meat is both healthier and better for the environment. Our sample came from a pool of self-selected participants, and there was an overrepresentation of individuals with a higher education and income than the UK average, especially in the Black and South Asian ethnic groups, although this was accounted for in the statistical analyses via survey weights. Lower-SEP respondents were less likely to agree with these statements compared to higher-SEP respondents in the overall sample, although this difference was only seen between the White respondents when each ethnic group was analyzed separately. Previous research has suggested that people in higher SEP groups may have more awareness about the negative environmental and health impact of meat consumption, as found in Grummon et al. (2022) and Taillie et al. (2022), which may be due to having had more education, or having more time and resources to access nutrition information (Clonan et al., 2016; Maguire & Monsivais, 2015). In the overall sample, men were also less likely to agree that overconsumption of red meat had a negative impact on the environment compared to women, and this difference was significant between South Asian men and women respondents, although differences within Black and White ethnic groups were not. A cluster analysis study examining reasonings for following vegetarian diets found that women were more likely to be motivated by environmental reasons than men (Trocchia & Janda, 2003). A qualitative study also found that being vegetarian was most strongly associated with altruistic values and the belief that eating meat was harmful to the environment, and more women than men expressed having these values and beliefs than men in that sample (Kalof et al., 1999). Gender-based socialization often lead women to value nurturing other living beings more than men, which may explain their higher levels of concern for the environment (Modlinska et al., 2020).

Importantly, we found that both South Asian and Black respondents were statistically significantly more likely than White respondents to agree that they were influenced by and wanted to eat similarly to friends and family. The stronger perceived influence of family and friends in minority ethnic groups in the UK may reflect the tradition of communal eating, sharing meals at social gatherings and celebrations and the higher value they place on friends and family (Chowbey & Harrop, 2016; Leung & Stanner, 2011). Minority ethnic groups are also more likely to live in multigenerational households, where one member of the family cooks for the whole household, and value the contribution of older generations in maintaining traditional eating practices (Cook et al., 2021). Frequently eating the same meals as a household may reframe food choices as not isolated and individual decisions, but as collective ones that are influenced by each member of the household and where compromise is needed to be reached and members of the household need to consume same or similar foods. Many minority ethnic groups in the UK come from collectivistic cultures which value conformity and following the majority’s behaviors and choices (Hofstede, 2011; Triandis, 2001). Collectivistic cultures also prioritize spending time with other members of society, creating more opportunities for individuals to influence each other. (Hofstede, 2011).

In the overall sample, higher SEP individuals were more likely to perceive friends and family to influence their food choices, and within ethnic groups, this difference was statistically significant for White respondents. Individuals with higher SEP might have more flexible work schedules and disposable income to have more occasions to share meals with their friends and family both at home and eating out, creating more opportunities to experience normative influences their close others have on them. Lower SEP occupations are more likely to require disruptive arrangements such as night shifts, isolated work, and limited breaks for food consumption, meaning that individuals with these occupations might choose and consume food away from their friends and family and without their influence.

### 4.1 Strengths and limitations

The present survey had the advantage of recruiting a large and ethnically diverse sample achieved through quota sampling from a pool of nationally representative respondents. To our knowledge, this survey is one of the first to provide a detailed insight into the norms, beliefs, and behaviours around meat consumption for White, South Asian and Black ethnic groups living in the UK.

All measures in the survey relied on self-reports, and actual behaviours of the respondents were not measured. However, since the focus of the study included exploring attitudes and perceptions of social norms (rather that identifying what the real prevalent norms were in society), self-report was an appropriate measure for the aims of this study. Additionally, the questions that are analyzed and presented here were asked to respondents as a part of a larger study that included questions about media usage, and sources of information used for food choices, therefore it is unlikely that a social desirability bias that would influence responses was introduced. The measures used for beliefs regarding the health and environmental impacts of red and processed meat consumption and those that measured perceived influence and support and desire to be similar to close friends and family were novel items developed for the purposes of this study. While the wording of each item was carefully deliberated on and decided on after reviewing measures from previous research, it should be noted that they are not previously validated measures.

The nature of the study also meant that data was only collected at a single time point. This cross-sectional design only provides a snapshot in time, and does not capture any trends or changes in attitudes, beliefs and behavior. It also is unable to reflect the effects of external events that may have influenced meat consumption behaviors, such as the COVID-19 pandemic and related restrictions. Future research can aim to have a repeated measures design and collect data at various timepoints in order to address this limitation.

The respondents were recruited among a pool of people that had already signed up to take part in similar research studies. This self-selection of individuals led to overrepresentation of higher educational attainment and social grade, especially among South Asian and Black respondents (given quotas for education and social grade were not set for these groups), which may impact the generalizability of our findings. We have aimed to account for this by using survey weights in our analysis, however, it should be noted that these weights reflected the YouGov panel (which is recruited and maintained to be nationally representative), but not the whole UK population.

Due to the hypothesis generating nature of our study on whether there were any differences between ethnic groups in their meat consumption at all, we did not correct the p-value threshold of statistical significance for multiple analyses. Without this correction, it could be suspected that the significance of 1 in 20 results could have occurred solely due to chance. We ran 60 regressions and 24 of them reached statistical significance, which roughly translates to 1 in 2.5 results, and these are unlikely to have occurred solely due to chance. We have reported the 95% confidence intervals and p-values of each regression to be open for interpretation and we advise the readers to take these into account when evaluating our findings.

Finally, for the data analysis, we grouped certain ethnic backgrounds together, which may have led to the disappearance of some nuances between different ethnicities. For example, Pakistani and Indian individuals were all analyzed in the South Asian ethnicity group while they may have different religious practices and food habits due to their Muslim-majority and Hindu-majority backgrounds respectively. Our participants also differed on whether they were born in Britain or else-where. Due to the size of our sample, we were not able to separately analyze those that were born in the UK and those that were not. We also did not collect any data on how strongly individuals identified with the culture of their birth country, which could have moderated their responses.

This study is an important first step in understanding differences in attitudes, beliefs, norms, and behaviors around meat consumption across the three largest ethnic groups in the UK. However, future research with bespoke study design and focused recruitment of specific ethnic, cultural, and religious subgroups is needed to better understand the dynamics of these differences and capture important nuances.

### 4.2 Implications

Eating is a social behavior and food choices can be influenced by normative expectations. Social norms-based interventions have been shown to be useful in promoting eating behaviors such as increased fruit and vegetable intake (Collins et al., 2019; Huitink et al., 2020; Mollen et al., 2013; Robinson et al., 2014; Thomas et al., 2017). However, recent literature has found mixed evidence regarding the effectiveness of social norm interventions for reducing meat consumption. While a dynamic social norm intervention reduced meat intake in online and university restaurant setting with US samples (Sparkman et al., 2020; Sparkman & Walton, 2017)), research from the UK that used an online study setting that measured intentions (Aldoh et al., 2021) and a real-life field trial that measured actual purchase data (Çoker, Pechey, et al., 2022) both found no effects of the intervention. The findings from this survey study offer a possible explanation since different ethnic groups in the UK have different perceptions of social influence from close others, which may moderate their responses to social norm-based interventions to change their eating behaviors. Tailoring interventions to different ethnic groups may increase their effectiveness, which has not yet been done at scale (Leung & Stanner, 2011). For example, for people who come from cultures that prioritize meal sharing and valuing social coherence, social norm-based interventions may be more effective. Other strategies may include intervening at the household or family level instead of targeting individuals to achieve increased adherence to the intervention. Intervention design can also keep in mind different cultural, social and religious values associated with food and diet when developing intervention materials, can involve community leaders in the co-creation process. Future research is needed to explore these hypotheses by testing interventions in various contexts and with diverse demographics.

## Figures and Tables

**Table 1:** Demographic characteristics of the study sample.

		White	South Asian	Black
Gender				
	Men	47.6%	45.8%	37.5%
	Women	52.4%	54.2%	62.5%
Social Grade^a^				
	ABC1	62.0%	74.4%	73.4%
	C2DE	38.0%	25.6%	26.6%
Age				
	18–24	7.9%	11.8%	10.5%
	25–34	16.6%	22.8%	25.8%
	35–44	18.9%	30.4%	23.1%
	45–54	15.9%	15.2%	16.2%
	55+	40.7%	19.9%	24.4%
Education^b^				
	Lower	46.0%	27.0%	26.8%
	Higher	54.0%	73.0%	73.2%
Marital Status				
	Married/civil partner	47.3%	55.6%	34.8%
	Living as married	15.4%	5.8%	11.5%
	Separated/divorced	8.0%	4.5%	8.4%
	Widowed	3.0%	1.9%	0.4%
	Never married	26.4%	32.3%	44.9%

a Social grades are a UK-specific socioeconomic status classification based on occupation. (A: upper middle class (higher managerial roles) B: middle middle class (lower managerial roles) C1: lower middle class (supervisory or clerical and junior managerial roles), C2: skilled working class (skilled manual laborers), D: working class (semi- and unskilled manual laborers), and E: non-working (pensioners and unemployed with state benefits)).

b Following the UK government’s education qualification levels for England, Wales and Northern Ireland and their Scottish equivalents, we categorized Level 1–4 (e.g. no formal qualifications, apprenticeships, certificates, GCSEs) into the “lower” education category and Levels 5–8 (e.g. diplomas of higher education, bachelor’s degrees, master’s degrees, doctorates of philosophy) into the “higher” category.

**Table 2:** Weighted proportions for self-reported meat consumption behaviors, intentions, beliefs, and perceived influence by ethnicity.

	White (N = 403)	South Asian (N = 382)	Black (N = 229)
***“Which, if any, of these best describes your usual eating habits?”***			
Vegan	2.3%	2.7%	0.7%
Vegetarian	7.3%	10.5%	2.7%
Pescatarian	1.5%	7.1%	4.1%
Flexitarian	11.7%	17.5%	12.9%
Meat Eater	73.7%	56.2%	70.8%
Other	3.6%	4.7%	8.0%
Don’t know	0.2%	0.5%	0.8%
***“Are you considering decreasing or increasing the amount of meat you eat?”***			
Recently decreased	23.4%	28.8%	26.4%
Wants to decrease	11.2%	18.1%	19.5%
No change, meat-eater	52.5%	30.4%	37.3%
No change, non-meat	11.2%	12.7%	7.0%
eater			
Wants to increase	0.3%	3.3%	5.8%
Recently increased	1.4%	6.8%	4.1%
***“Eating less red and processed meat is better for the environment”***			
Strongly agree	27.9%	35.5%	27.0%
Tend to agree	35.4%	32.7%	34.5%
Don’t Know	16.5%	15.1%	23.5%
Tend to disagree	12.0%	11.3%	6.5%
Strongly disagree	8.2%	5.4%	8.5%
***“Eating less red and processed meat is healthier”***			
Strongly agree	27.8%	34.7%	38.1%
Tend to agree	43.3%	39.8%	36.2%
Don’t Know	10.6%	10.5%	8.7%
Tend to disagree	12.3%	9.2%	9.9%
Strongly disagree	6.0%	5.8%	7.1%
***“My close friends and family would support me if I decided to reduce my meat*** ***consumption”***			
Strongly agree	27.4%	32.5%	39.1%
Tend to agree	42.4%	40.0%	36.2%
Don’t Know	20.3%	16.0%	17.0%
Tend to disagree	5.7%	8.8%	5.5%
Strongly disagree	4.1%	2.7%	2.2%
***“My close friends and family influence my food choices”***			
Strongly agree	2.6%	10.2%	10.1%
Tend to agree	19.3%	33.3%	26.8%
Don’t Know	5.0%	8.9%	8.7%
Tend to disagree	32.9%	26.7%	26.3%
Strongly disagree	40.3%	21.0%	28.2%
***“It matters to me to eat similar foods to my close friends and family”***			
Strongly agree	3.1%	9.0%	9.4%
Tend to agree	12.8%	29.0%	18.2%
Don’t Know	5.1%	9.7%	13.1%
Tend to disagree	35.8%	31.1%	27.9%
Strongly disagree	43.3%	21.3%	31.4%

**Table 3:** Weighted proportions of self-reported meat consumption behaviors, intentions, beliefs, and perceived influence by ethnicity and gender.

		White			South Asian			Black		
		Men (N = 192)	Women (N = 211)		Men (N = 175)	Women (N = 207)		Men (N = 86)	Women (N = 143)
***“Which, if any, of these best describes your usual eating habits?”***									
Vegan		1.0%	3.5%		2.3%	3.1%		0%	1.3%
Vegetarian		4.0%	10.4%		6.2%	14.6%		1.9%	3.5%
Pescatarian		0.5%	1.8%		8.1%	7.8%		4.9%	3.3%
Flexitarian		8.7%	14.6%		16.4%	18.6%		10.9%	14.7%
Meat Eater		81.6%	66.2%		59.9%	52.7%		75.1%	67.1%
Other		3.8%	3.5%		6.7%	2.6%		7.2%	8.7%
Don’t know		0.5%	0%		0.4%	0.6%		0%	1.4%
***“Are you considering decreasing or increasing the amount of meat you eat?*** *”*									
Recently decreased		18.7%	27.7%		27.6%	29.9%		22.4%	30.0%
Wants to decrease		11.6%	10.8%		5.0%	11.5%		16.0%	22.7%
No change meat		62.9%	42.8%		31.9%	29.0%		42.3%	32.8%
No change no meat		5.9%	1.6%		9.6%	15.6%		6.4%	7.5%
Wants to increase		0.0%	0.7%		1.9%	4.6%		10.0%	2.0%
Recently increased		0.9%	1.9%		4.0%	9.4%		3.0%	5.1%
***“Eating less red and processed meat is better for the environment”***									
Strongly agree		19.4%	35.8%		22.5%	48.0%		22.8%	30.5%
Tend to agree		42.1%	29.1%		38.1%	27.5%		31.7%	36.9%
Don’t Know		15.9%	17.1%		16.7%	13.5%		25.5%	21.8%
Tend to disagree		11.6%	12.4%		17.7%	5.2%		9.2%	4.2%
Strongly disagree		11.0%	5.6%		5.0%	5.8%		10.8%	6.6%
***“Eating less red and processed meat is healthier”***									
Strongly agree		21.1%	34.1%		28.1%	41.2%		22.8%	30.5%
Tend to agree		45.9%	40.9%		44.3%	35.4%		31.7%	36.9%
Don’t Know		10.9%	10.4%		10.8%	10.3%		25.5%	21.8%
Tend to disagree		14.3%	10.4%		12.8%	5.6%		9.2%	4.2%
Strongly disagree		7.8%	4.2%		4.1%	7.5%		10.8%	6.6%
***“My close friends and family would support me if I decided to reduce my meat consumption”***									
Strongly agree		20.5%	34.0%		30.1%	34.9%		36,6%	41.3%
Tend to agree		45.3%	39.7%		42.1%	37.8%		31.8%	40.1%
Don’t Know		22.9%	17.9%		12.3%	19.6%		24.1%	10.8%
Tend to disagree		7.3%	4.2%		13.3%	4.5%		5.3%	5.7%
Strongly disagree		4.1%	4.2%		2.2%	3.2%		2.2%	2.2%
***“My close friends and family influence my food choices”***									
Strongly agree		3.3%	1.9%		12.3%	8.1%		8.4%	11.5%
Tend to agree		20.8%	17.8%		30.2%	36.4%		24.9%	28.4%
Don’t Know		4.6%	5.4%		8.2%	9.5%		14.5%	3.7%
Tend to disagree		36.0%	30.0%		26.7%	26.7%		33.2%	20.3%
Strongly disagree		35.3%	44.9%		22.7%	19.3%		19.0%	36.1%
***“It matters to me to eat similar foods to my close friends and family”***									
Strongly agree		3.2%	3.1%		8.3%	9.5%		9.6%	9.3%
Tend to agree		12.3%	13.2%		27.1%	30.9%		21.2%	15.6%
Don’t Know		4.0%	6.0%		10.8%	8.7%		17.9%	8.8%
Tend to disagree		40.2%	31.7%		32.4%	29.9%		26.6%	29.1%
Strongly disagree		40.3%	46.0%		21.5%	21.0%		24.6%	37.2%

**Table 4:** Weighted proportions for meat consumption behaviors, intentions, beliefs, and perceived influence by ethnicity and social grade.

	White		South Asian		Black	
	ABC1 (N = 250)	C2DE (N = 153)	ABC1 (N = 284)	C2DE (N = 98)	ABC1 (N = 168)	C2DE (N = 61)
***“Which, if any, of these best describes your usual eating habits?”***						
Vegan	3.7%	0.4%	3.1%	1.9%	0.6%	0.9%
Vegetarian	8.1%	6.2%	12.7%	6.0%	2.8%	2.7%
Pescatarian	2.1%	0.0%	9.9%	4.0%	2.3%	7.2%
Flexitarian	13.0%	10.0%	19.5%	13.7%	14.2%	10.8%
Meat Eater	67.6%	81.7%	52.2%	64.1%	68.8%	74.3%
Other	5.5%	1.1%	2.1%	9.7%	11.0%	2.7%
Don’t know	0.0%	0.5%	0.4%	0.7%	0.4%	1.4%
***“Are you considering decreasing or increasing the amount of meat you eat?”***						
Recently decreased	24.4%	22.0%	24.6%	38.1%	28.7%	22.4%
Wants to decrease	11.8%	10.4%	16.4%	22.2%	18.1%	22.0%
No change meat	48.7%	57.6%	29.8%	31.8%	35.9%	39.7%
No change no meat	14.0%	7.4%	17.3%	2.2%	6.8%	7.4%
Wants to increase	0.0%	0.8%	4.7%	0.0%	5.2%	6.8%
Recently increased	1.2%	1.7%	7.2%	5.8%	5.5%	1.7%
***“Eating less red and processed meat is better for the environment”***						
Strongly agree	30.8%	23.9%	41.7%	23.2%	27.1%	26.7%
Tend to agree	37.8%	32.2%	30.5%	36.9%	36.2%	31.6%
Don’t Know	13.6%	20.5%	13.0%	19.2%	19.5%	30.4%
Tend to disagree	13.2%	10.5%	8.2%	17.6%	5.6%	8.1%
Strongly disagree	4.6%	13.0%	6.6%	3.1%	11.6%	3.2%
***“Eating less red and processed meat is healthier”***						
Strongly agree	28.4%	27.0%	41.4%	21.7%	41.7%	31.8%
Tend to agree	48.7%	36.2%	39.5%	40.2%	27.0%	52.3%
Don’t Know	7.9%	14.2%	8.8%	13.9%	9.9%	6.5%
Tend to disagree	10.4%	14.8%	5.3%	16.8%	12.1%	6.1%
Strongly disagree	4.5%	7.8%	5.0%	7.4%	9.3%	3.3%
***“My close friends and family would support me if I decided to reduce my meat consumption”***						
Strongly agree	29.5%	24.8%	37.0%	23.7%	38.8%	39.7%
Tend to agree	46.9%	36.5%	36.0%	47.5%	40.5%	28.8%
Don’t Know	13.5%	29.4%	15.0%	18.0%	10.1%	29.1%
Tend to disagree	6.8%	4.2%	8.8%	8.9%	8.0%	1.1%
Strongly disagree	3.4%	5.1%	3.2%	1.8%	2.6%	1.4%
***“My close friends and family influence my food choices”***						
Strongly agree	3.4%	1.5%	13.6%	3.4%	12.2%	6.4%
Tend to agree	22.7%	14.7%	32.9%	34.2%	31.6%	18.5%
Don’t Know	2.7%	8.0%	6.3%	13.9%	1.8%	20.7%
Tend to disagree	35.2%	35.2%	25.8%	28.4%	29.7%	20.3%
Strongly disagree	36.0%	46.0%	21.4%	20.1%	24.8%	34.1%
***“It matters to me to eat similar foods to my close friends and family”***						
Strongly agree	4.3%	1.5%	10.8%	5.1%	12/3%	4.4%
Tend to agree	16.4%	7.9%	29.5%	28.2%	19.3%	16.3%
Don’t Know	4.4%	5.9%	5.3%	18.4%	6.6%	24.3%
Tend to disagree	40.5%	29.5%	30.8%	31.8%	32.5%	19.9%
Strongly disagree	34.3%	55.2%	23.7%	16.5%	29.2%	35.1%

## Data Availability

The data that support the findings of this study are openly available in OSF at https://osf.io/zuytq.

## Data availability

OSF link for data included in manuscript

## References

[R1] Aldoh A, Sparks P, Harris PR (2021). Dynamic norms and food choice: Reflections on a failure of minority norm information to influence motivation to reduce meat consumption. Sustainability.

[R2] Chowbey P, Harrop D (2016). Healthy eating in UK minority ethnic households: Influences and way forward.

[R3] Cialdini RB, Reno RR, Kallgren CA (1990). A focus theory of normative conduct - recycling the concept of norms to reduce littering in public places. Journal of Personality and Social Psychology.

[R4] Clonan A, Roberts KE, Holdsworth M (2016). Socioeconomic and demographic drivers of red and processed meat consumption: Implications for health and environmental sustainability. Proceedings of the Nutrition Society.

[R5] Çoker EN, Jebb SA, Stewart C, Clark M, Pechey R (2022). Perceptions of social norms around healthy and environmentally-friendly food choices: Linking the role of referent groups to behavior. Frontiers in Psychology.

[R6] Çoker EN, Pechey R, Frie K, Jebb SA, Stewart C, Higgs S, Cook B (2022). A dynamic social norm messaging intervention to reduce meat consumption: A randomized cross-over trial in retail store restaurants. Appetite.

[R7] Collins EI, Thomas JM, Robinson E, Aveyard P, Jebb SA, Herman CP, Higgs S (2019). Two observational studies examining the effect of a social norm and a health message on the purchase of vegetables in student canteen settings. Appetite.

[R8] Cook EJ, Powell FC, Ali N, Penn-Jones CP, Ochieng B, Constantinou G, Randhawa G (2021). ‘They are kids, let them eat’: A qualitative investigation into the parental beliefs and practices of providing a healthy diet for young children among a culturally diverse and deprived population in the UK. International Journal of Environmental Research and Public Health.

[R9] De Backer C, Erreygers S, De Cort C, Vandermoere F, Dhoest A, Vrinten J, Van Bauwel S (2020). Meat and masculinities. Can differences in masculinity predict meat consumption, intentions to reduce meat and attitudes towards vegetarians?. Appetite.

[R10] de Boer J, Schösler H, Aiking H (2017). Towards a reduced meat diet: Mindset and motivation of young vegetarians, low, medium and high meat-eaters. Appetite.

[R11] Ekmekcioglu C, Wallner P, Kundi M, Weisz U, Haas W, Hutter HP (2018). Red meat, diseases, and healthy alternatives: A critical review. Critical Reviews in Food Science and Nutrition.

[R12] Ellithorpe ME, Takahashi B, Alumit Zeldes G, Dorrance-Hall E, Chavez M, Plasencia J (2022). Family and cultural perceptions about meat consumption among hispanic/latino and white adults in the United States. Ecology of Food and Nutrition.

[R13] Fehr E, Fischbacher U (2004). Social norms and human cooperation. Trends in Cognitive Sciences.

[R14] Gal D, Wilkie J (2010). Real men don’t eat quiche: Regulation of gender-expressive choices by men. Social Psychological and Personality Science.

[R15] Gerber PJ, Steinfeld H, Henderson B, Mottet A, Opio C, Dijkman J, Falcucci A, Tempio G (2013). Tackling climate change through livestock: A global assessment of emissions and mitigation opportunities.

[R16] Gilbert PA, Khokhar S (2008). Changing dietary habits of ethnic groups in Europe and implications for health. Nutrition Reviews.

[R17] Gonçalves D, Coelho P, Martinez LF, Monteiro P (2021). Nudging consumers toward healthier food choices: A field study on the effect of social norms. Sustainability.

[R18] Graça J, Godinho CA, Truninger M (2019). Reducing meat consumption and following plant-based diets: Current evidence and future directions to inform integrated transitions. Trends in Food Science & Technology.

[R19] Grummon AH, Goodman D, Jaacks LM, Taillie LS, Chauvenet CA, Salvia MG, Rimm EB (2022). Awareness of and reactions to health and environmental harms of red meat among parents in the United States. Public Health Nutrition.

[R20] Hamlett J, Bailey AR, Alexander A, Shaw G (2008). Ethnicity and consumption: South asian food shopping patterns in Britain, 1947—75 1. Journal of Consumer Culture.

[R21] Hartmann C, Siegrist M (2017). Consumer perception and behaviour regarding sustainable protein consumption: A systematic review. Trends in Food Science & Technology.

[R22] Hinrichs K, Hoeks J, Campos L, Guedes D, Godinho C, Matos M, Graça J (2022). Why so defensive? Negative affect and gender differences in defensiveness toward plant-based diets. Food Quality and Preference.

[R23] Hofstede G (2011). Dimensionalizing cultures: T he hofstede model in context. Online readings in psychology and culture.

[R24] Huitink M, Poelman MP, van den Eynde E, Seidell JC, Dijkstra SC (2020). Social norm nudges in shopping trolleys to promote vegetable purchases: A quasi-experimental study in a supermarket in a deprived urban area in The Netherlands. Appetite.

[R25] Kalof L, Dietz T, Stern PC, Guagnano GA (1999). Social psychological and structural influences on vegetarian beliefs. Rural Sociology.

[R26] Leahy E, Lyons S, Tol RS (2010). National determinants of vegetarianism.

[R27] Leung G, Stanner S (2011). Diets of minority ethnic groups in the UK: Influence on chronic disease risk and implications for prevention. Nutrition Bulletin.

[R28] Macdiarmid JI, Douglas F, Campbell J (2016). Eating like there’s no tomorrow: Public awareness of the environmental impact of food and reluctance to eat less meat as part of a sustainable diet. Appetite.

[R29] Maguire ER, Monsivais P (2015). Socio-economic dietary inequalities in UK adults: An updated picture of key food groups and nutrients from national surveillance data. British Journal of Nutrition.

[R30] Modlinska K, Adamczyk D, Maison D, Pisula W (2020). Gender differences in attitudes to vegans/vegetarians and their food preferences, and their implications for promoting sustainable dietary patterns–a systematic review. Sustainability.

[R31] Mollen S, Rimal RN, Ruiter RA, Kok G (2013). Healthy and unhealthy social norms and food selection. Findings from a field-experiment. Appetite.

[R32] Papier K, Fensom GK, Knuppel A, Appleby PN, Tong TY, Schmidt JA, Travis RC, Key TJ, Perez-Cornago A (2021). Meat consumption and risk of 25 common conditions: Outcome-wide analyses in 475,000 men and women in the UK biobank study. BMC Medicine.

[R33] Piazza J, Ruby MB, Loughnan S, Luong M, Kulik J, Watkins HM, Seigerman M (2015). Rationalizing meat consumption. The 4Ns. Appetite.

[R34] Robinson E, Harris E, Thomas J, Aveyard P, Higgs S (2013). Reducing high calorie snack food in young adults: A role for social norms and health based messages. International Journal of Behavioral Nutrition and Physical Activity.

[R35] Robinson Thomas J, Aveyard P, Higgs S (2014). What everyone else is eating: A systematic review and meta-analysis of the effect of informational eating norms on eating behavior. Journal of the Academy of Nutrition and Dietetics.

[R36] Rosenfeld DL, Tomiyama AJ (2021). Gender differences in meat consumption and openness to vegetarianism. Appetite.

[R37] Rothgerber H (2013). Real men don’t eat (vegetable) quiche: Masculinity and the justification of meat consumption. Psychology of Men and Masculinity.

[R38] Rozin P, Hormes JM, Faith MS, Wansink B (2012). Is meat male? A quantitative multimethod framework to establish metaphoric relationships. Journal of Consumer Research.

[R39] Ruby MB, Heine SJ (2011). Meat, morals, and masculinity. Appetite.

[R40] Sanchez-Sabate R, Sabaté J (2019). Consumer attitudes towards environmental concerns of meat consumption: A systematic review. International Journal of Environmental Research and Public Health.

[R41] Scarborough P, Clark M, Cobiac L, Papier K, Knuppel A, Lynch J, Harrington R, Key T, Springmann M (2023). Greenhouse gas emissions, land use, water use, water pollution and biodiversity impact of vegans, vegetarians, fish-eaters and meat-eaters in the UK. Nature Food.

[R42] Schösler H, de Boer J, Boersema JJ, Aiking H (2015). Meat and masculinity among young Chinese, Turkish and Dutch adults in The Netherlands. Appetite.

[R43] Sparkman G, Walton GM (2017). Dynamic norms promote sustainable behavior, even if it is counternormative. Psychological Science.

[R44] Sparkman G, Weitz E, Robinson TN, Malhotra N, Walton GM (2020). Developing a scalable dynamic norm menu-based intervention to reduce meat consumption. Sustainability.

[R45] Stewart C, Piernas C, Cook B, Jebb SA (2021). Trends in UK meat consumption: Analysis of data from years 1-11 (2008-09 to 2018-19) of the national diet and nutrition survey rolling programme. The Lancet Planetary Health.

[R46] Stok FM, de Vet E, de Ridder DT, de Wit JB (2016). The potential of peer social norms to shape food intake in adolescents and young adults: A systematic review of effects and moderators. Health Psychology Review.

[R47] Taillie LS, Prestemon CE, Hall MG, Grummon AH, Vesely A, Jaacks LM (2022). Developing health and environmental warning messages about red meat: An online experiment. PLoS One.

[R48] Thomas JM, Ursell A, Robinson EL, Aveyard P, Jebb SA, Herman CP, Higgs S (2017). Using a descriptive social norm to increase vegetable selection in workplace restaurant settings. Health Psychology.

[R49] Triandis HC (2001). Individualism-collectivism and personality. Journal of Personality.

[R50] Trocchia PJ, Janda S (2003). A cluster analytic approach for consumer segmentation using the vegetarian/meatarian distinction. Journal of Food Products Marketing.

[R51] UKGovernment List of ethnic groups.

[R52] van Bussel L, Kuijsten A, Mars M, van’t Veer P (2022). Consumers’ perceptions on food-related sustainability: A systematic review. Journal of Cleaner Production.

[R53] Willett W, Rockström J, Loken B, Springmann M, Lang T, Vermeulen S, Garnett T, Tilman D, DeClerck F, Wood A (2019). Food in the anthropocene: The EAT–lancet commission on healthy diets from sustainable food systems. The Lancet.

